# Comparison of mini-open reduction and autologous bone grafting with closed reduction and intramedullary device insertion for tibial shaft fractures: a retrospective study

**DOI:** 10.1186/s13018-023-04024-9

**Published:** 2023-07-22

**Authors:** Danfeng Xu, Jianxin Xie, Bing Wu, Yubin Zou, Yong He, Zhaosheng Li

**Affiliations:** 1 Department of Spine Surgery, The Central Hospital Affiliated to Shaoxing University, Hua-yu Road 1, Keqiao, Shaoxing, 312030 People’s Republic of China; 2 Central Laboratory, The Central Hospital Affiliated to Shaoxing University, Shaoxing, 312030 People’s Republic of China

**Keywords:** Tibial shaft fractures, Intramedullary nailing, Open reduction, Closed reduction, Autologous bone grafting

## Abstract

**Background:**

We compared the clinical efficacy of mini-open reduction and autologous bone grafting (*G*_M_) and closed reduction (*G*_C_) using intramedullary nailing for the treatment of tibial shaft fractures.

**Methods:**

This retrospective study included 70 tibial shaft fractures treated with *G*_M_ or *G*_C_ between January 2018 and December 2021. The demographic characteristics and clinical outcomes were compared between the two treatment methods.

**Results:**

This study included 70 patients who were followed-up for 12.4 months. In total, 31 and 39 patients were treated with *G*_M_ and *G*_C_, respectively. The operative duration was significantly shorter for *G*_M_ (95.2 ± 19.3 min) than for *G*_C_ (105.5 ± 22.2 min, *p* = 0.0454). The number of radiation times was significantly lower for *G*_M_ (14.7 ± 6.3) than for *G*_C_ (22.2 ± 9.2, *p* < 0.005). There were no statistically significant differences between the groups in terms of the wound complication or infection rates. The malunion and nonunion rates were high after *G*_C_ than after *G*_M_, but there are no significant differences between the groups.

**Conclusions:**

Closed reduction and intramedullary nailing remains the first choice for tibial shaft fractures. *G*_M_ is a safe and effective treatment worth considering. Future prospective randomized controlled trials are warranted.

## Introduction

Tibial fractures result from high- and low-energy trauma [1]. The aim of surgical treatment is to promote early postoperative weight-bearing and rehabilitation. The treatments for tibial fractures include intramedullary nailing (IMN), plating, and external fixation [2, 3]. Multiple studies have evaluated the safety and effectiveness of IMN, and have found that it promotes bone healing, early mobilization, and return to function. IMN is commonly performed and effective for the treatment of tibial fractures [4–7]. Tibial shaft fractures may be treated with IMN for internal fixation using limited open reduction of the fractured point [8, 9] and implanting a bone graft harvested from the opening point of the tibial tuberosity to the fractured end [10]. This method is associated with better reduction, fewer soft tissue operations and X-rays, shortened reduction time, and improved fracture healing rate compared to closed reduction. We retrospectively compared the outcomes of mini-open reduction and autologous bone grafting followed by IMN (*G*_M_) and closed reduction (*G*_C_) for the treatment of closed tibial fractures. We hypothesized that the former is associated with reduced operation time and improved fracture healing rate without an increased risk of complications.

## Methods

### Patients

This retrospective study enrolled patients with displaced closed fracture of the tibial shaft who were treated with IMN and presented for regular follow-up for ≥ 6 months or until fracture union between January 2018 and December 2021 at the Department of our hospital. Indications for open reduction include cases where a satisfactory closed reduction could not be achieved or when there is the presence of intramedullary cortical bone debris. Ethical approval was obtained from the Ethics Committee of our hospital. We excluded patients with open or pathological fractures, compartment syndrome, infection, or concomitant diseases. In total, 70 patients with tibial fractures were enrolled. The demographic characteristics of the study variables are presented in Table 1.

**Table 1:** Patient characteristics data

	*G*_M_	*G*_C_	*χ*^2^ or *t* value	*p*-value
Gender (male/female)	24/7	27/12	0.59	0.444
Mean age, years	38.9 ± 12.2	41.9 ± 13.9	0.96	0.340
Fracture side (left/right)	17/14	19/20	0.26	0.611
Follow-up time (month)	12.6 ± 4.2	12.2 ± 3.8	0.42	0.676
Mechanism of injury			0.32	0.956
Traffic	18	21		
Falling	7	11		
Sports	2	2		
Others	4	5		
OTA classification			0.48	0.788
42-A	20	22		
42-B	7	11		
42-C	4	6		

### Surgical methods

The surgery was performed by a chief surgeon or associate chief surgeon.

#### Observation group (GM)

**Fig. 1:**
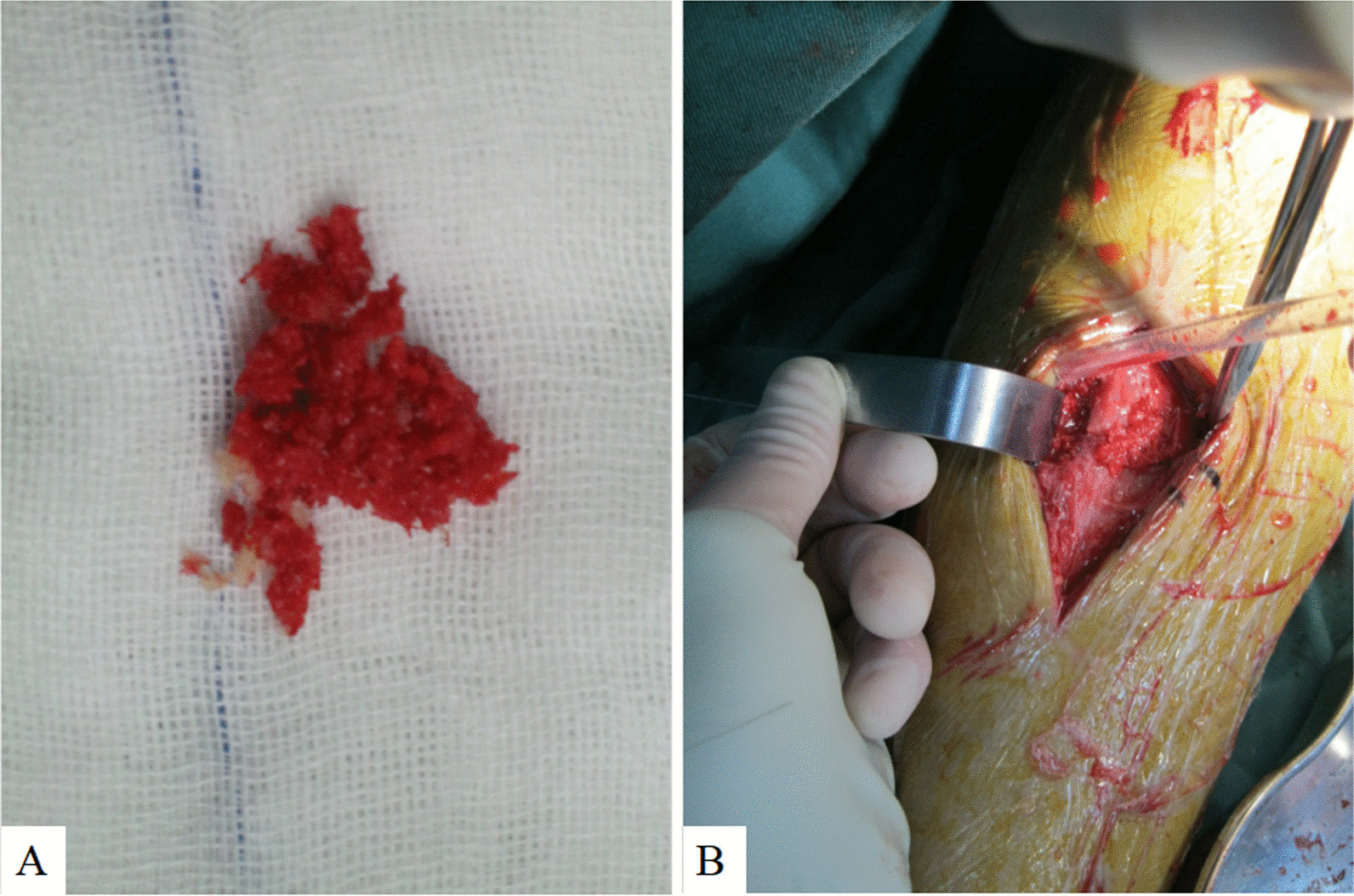
**A** showed the bone harvested in the procedure. **B** showed the small incision which was helpful for fracture reduction and autologous bone grafting

After anesthesia, the patient was placed in supine position on a fluoroscopic surgical bed and a balloon tourniquet was applied. A longitudinal incision 4–5 cm long was made below the patella. The patellar ligament was incised longitudinally, and the point of entry for the tibial IMN was determined. The tibial tuberosity was opened approximately 0.5 cm posteromedial to the tibial tuberosity using a bone awl, and free bone fragments were selected as the opening point (Fig. 1A). A small auxiliary incision of approximately 3–4 cm was made at the displaced end of the bone to reposition and fix the fractured segment using bone-holding forceps (Fig. 1B). After insertion of the guide wire and satisfactory fluoroscopic reduction, the medullary canal was reamed to collect a sample. The medullary canal was expanded to match the IMN diameter. The IMN was inserted from the proximal tibia to lock the distal and proximal ends of the bone. C-arm X-ray was performed to visualize the fracture site, screw length, and anatomical reduction. The harvested bone was grafted onto the fractured site (Fig. 1B). The incision was irrigated and closed in layers.

#### Control group (GC)

Conventional closed reduction and IMN was performed.

#### Postoperative management

Postoperative antibiotics were administered routinely for 24–48 h to prevent infection. The patients were advised to perform functional exercises from three days after the surgery.

### Observational indices

We recorded the operative duration, radiation time, intraoperative blood loss, and postoperative complications, such as nonunion, infection, and malunion. Malunion is characterized by an excess of 5° of deviation in any plane, 15° of internal rotation, 20° of external rotation, a foreshortening exceeding 1 cm, or over 50% dislocation involving any fracture site.

### Statistical analysis

Data were analyzed using SPSS software (version 22.0; IBM Corp., Armonk, NY, USA). Continuous variables are presented as means ± standard deviations and were analyzed using an independent samples t test. Qualitative data were analyzed using the Chi-square test. *P* < 0.05 was considered to be statistically significant.

## Results

This study enrolled 70 patients with a mean age of 40.6 years; 31 were treated with *G*_M_ and 39 were treated with *G*_C_. The patients were followed-up for 12.4 months. The characteristics of the study participants are presented in Table 1.

### Surgical outcomes

**Fig. 2:**
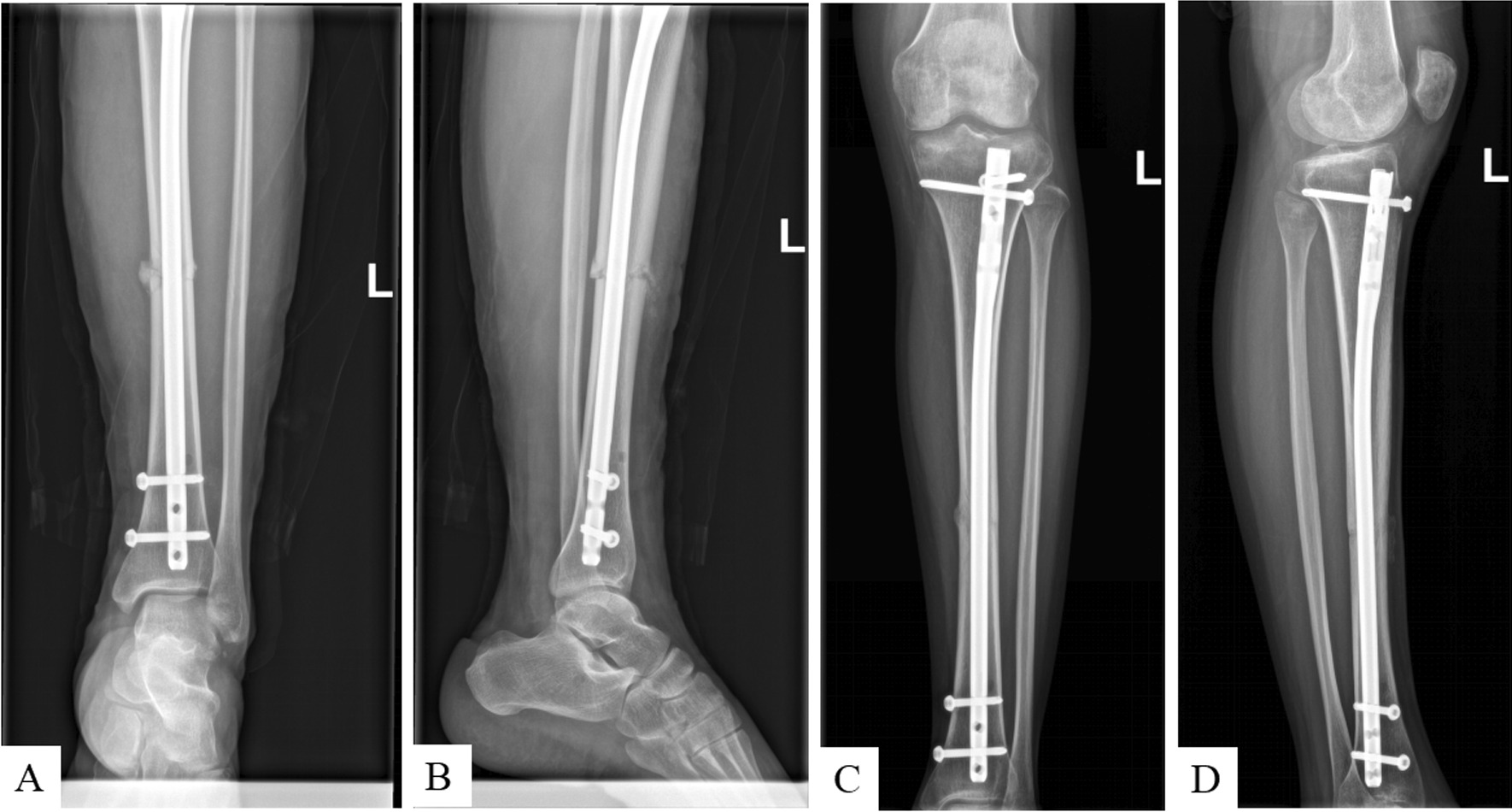
Postoperative X-rays in *G*_M_. **A** and **B** were 1 day postoperative X-rays. A perfectly fracture reduction was achieved. **B** Bone grafting shadow was visible. **C** and **D** were 6 months postoperative X-rays. The fracture was healing well

The operative duration was significantly shorter for *G*_M_ (95.2 ± 19.3 min) than for *G*_C_ (105.5 ± 22.2 min, *p* = 0.0454). The number of radiation times were significantly lower for *G*_M_ (14.7 ± 6.3) than for *G*_C_ (22.2 ± 9.2, *p* < 0.005). The intraoperative blood losses were 46.8 ± 28.6 ml and 49.5 ± 25.2 ml in the *G*_M_ and *G*_C_ groups, respectively, with no significant differences between the groups (Table 2). Figure 2 shows postoperative X-rays in *G*_M_. A perfectly fracture reduction was achieved and the fracture was healing well.

**Table 2:** Comparison of surgical outcomes in two treatment groups

	*G*_M_	*G*_C_	*t* value	*p*-value
Operative duration (min)	95.2 ± 19.3	105.5 ± 22.2	2.04	0.0454
Number of radiation times	14.7 ± 6.3	22.2 ± 9.2	3.84	0.0003
Intraoperative blood loss (ml)	46.8 ± 28.6	49.5 ± 25.2	0.42	0.675

### Postoperative complications

No statistically significant differences were observed in the wound complication or infection rate between the groups. Malunion and nonunion were common in the *G*_C_ group than in the *G*_M_ group, but there are no significant differences between the groups (Table 3).

**Table 3:** Comparison of complications in two treatment groups

	*G*_M_	*G*_C_	*χ*^2^ value	*p*-value
Infection (%)	0	0		
Nonunion (%)	0	2(5.1)	1.64	0.201
Malunion (%)	0	3(7.7)	2.49	0.114

## Discussion

We compared the safety and efficacy of *G*_M_ and *G*_C_ for the treatment of tibial shaft fractures. There were significant differences between the groups in terms of the operative duration and the number of radiation times. Besides low malunion and nonunion rates were found in *G*_M_, indicating favorable results of *G*_M_ compared to *G*_C_.

Tibial fractures are common and have been treated using several different methods. Malunion, nonunion, and wound infection are common postoperative complications after the treatment of tibial fractures [11]. IMN is performed for most cases of tibial fractures because it is minimally invasive, avoids soft tissue stripping, causes less bleeding, and preserves the vascular supply [6, 7]. However, it is associated with a high rate of malunion [12]. During closed reduction, it is difficult to reestablish the appropriate tibial length, alignment, and rotation before inserting the guidewire, thereby necessitating frequent X-ray use. In addition, the soft tissues near the fracture site are damaged during multiple reduction attempts [13]. Several studies have evaluated the effectiveness of open reduction and IMN for the treatment of tibial shaft fractures. Bishop et al. [8] found that open reduction through a small incision with careful soft tissue manipulation was safe and effective. It was associated with high-quality reduction, which promoted fracture healing. There were no significant differences in the nonunion or infection rates between this technique and closed reduction. Our results are in line with a retrospective study [9] that found similar outcomes between open and closed reduction. Open reduction significantly reduces the operation time and improves patient satisfaction. The operation time and the number of radiation times were significantly shorter and lower with *G*_M_ than with *G*_C_. There were no significant differences in the intraoperative blood loss between the two groups, which may be explained by the use of a tourniquet. *G*_M_ was associated with improved fracture healing and low malunion rate.

Although most tibial shaft fractures are treated successfully, nonunion is a common complication because of small muscle tissue attached to the distal tibia and insufficient blood supply. The nonunion rate after tibial fractures is 5–17% [14–16]. Autologous bone grafting is the gold-standard treatment for nonunion [17]. IMN is associated with excessive removal of bone at the point of nail insertion during reaming. We collected the excess bone and implanted it onto the fracture site to promote fracture healing. Autologous bone grafting improves the local biological factors at the fracture site to promote healing. During *G*_M_, bone harvesting was convenient and required no additional incision or costs. Bone grafts are usually obtained from the iliac crest, which requires an additional procedure [18]. Other implants that promote fracture healing, such as bone morphogenetic proteins [19] and platelet-rich plasma [20], are associated with additional costs [21, 22]. However, *G*_M_ overcomes these problems. The small incision at the fracture site allowed adequate fracture reduction and alignment, as well as bone grafting to promote fracture healing without the need for an additional procedure (Fig. 2). As a result, there is none nonunion after *G*_M_.

In the present study, we did not record fracture healing time as an outcome. Patients generally present for follow-up visits at almost 1 month postoperatively, which made it difficult to determine the fracture healing time accurately. To reduce the influence of confounding factors, we included patients with only closed fractures and excluded patients with open or pathological fractures, compartment syndrome, infection, or severe concomitant disease.

This study had several limitations. First, this was a retrospective study with a short follow-up duration. Second, we enrolled a small number of participants and did not evaluate their functional outcomes. Third, we excluded patients with open fractures. Therefore, it is unclear whether bone harvesting on open fracture sites reduce the risk of infection and other complications.

In conclusion, *G*_M_ is safe and effective for the treatment of tibial shaft fracture. While closed reduction and intramedullary nailing continue to be the preferred approach for managing tibial shaft fractures, the utilization of mini-open reduction combined with autologous bone grafting may be taken into consideration when closed techniques are unsuccessful. Future prospective randomized controlled trials are warranted.
